# Adverse Events with Hypoglossal Nerve Stimulation in the Treatment of Obstructive Sleep Apnea—A Systematic Review of Clinical Trials and Real-World Data

**DOI:** 10.3390/jcm13154282

**Published:** 2024-07-23

**Authors:** Mathias Wollny, Clemens Heiser, Ulrich Sommer, Christoph Schöbel, Marcel Braun

**Affiliations:** 1 MedImbursement, 27412 Tarmstedt, Germany; 2 Department of Otorhinolaryngology/Head and Neck Surgery, Klinikum rechts der Isar, Technical University of Munich, 80539 München, Germany; 3 ENT-Center Mangfall-Inn, 83043 Bad Aibling, Germany; 4 Translational Neurosciences, Faculty of Medicine and Health Sciences, University of Antwerp, 2610 Antwerp, Belgium; 5 Department of Pneumology, University Medicine Essen—Ruhrlandklinik, West German Lung Center, University Duisburg-Essen, 45141 Essen, Germany; 6 Faculty of Sleep and Telemedicine, University Medicine Essen—Ruhrlandklinik, West German Lung Center, University Duisburg-Essen, 45141 Essen, Germany

**Keywords:** neurostimulation, sleep apnea, complications, patient experience

## Abstract

**Background/Objectives:** Hypoglossal nerve stimulation (HNS) emerged as an alternative treatment for patients with obstructive sleep apnea (OSA) a decade ago. Long-term clinical trials and real-world data show that HNS treatment provides significant and sustained improvements in both OSA disease control and quality-of-life measures over time. Given the nature of HNS treatment, with the requirement of using an implantable neurostimulation system, patient safety is a critical domain in the assessment of this technology. The objective of this review was to evaluate adverse events (AEs) and complications with HNS therapy in a systematic review of published evidence. **Methods:** Medline, Cochrane, and Web of Science were systematically searched to identify randomized controlled and real-world observational studies reporting relevant outcomes with HNS therapy for treatment of OSA that included procedure-, device-, and treatment-related AEs. **Results:** Out of 418 articles screened, 27 were reviewed for eligibility, and 17 studies, the majority found to have low-to-moderate risk of bias, with data on 1962 patients were included for further analysis. Across included studies, reporting of AEs was heterogeneous with regard to the classifications used and the extent of reporting. Over an average follow-up duration of 17.5 ± 16.9 months, the pooled mortality rate was 0.01% (95% CI = 0.0 to 0.2%), with all reported deaths being unrelated to HNS treatment. The HNS system survival probability over the follow-up time of 60 months was 0.9834 (95% CI = 0.9768 to 0.9882), with infections and request for removal by patients being the most common indications. The pooled surgical revision rate was 0.08% (95% CI 0.0 to 0.2%). Most reported treatment-related side effects were transient stimulation-related discomfort (0.08%, 95% CI = 0.0 to 0.2%) and tongue abrasions (0.07%, 95% CI = 0.0 to 0.2%). Based on the systematic review, a standardized set of endpoints was defined, aiming to harmonize safety data relevant to HNS therapy. **Conclusions:** In this systematic review, HNS therapy for treatment of OSA is associated with a positive patient safety profile. AEs occur mainly at device implantation and during the treatment acclimatization period. Due to a lack of available evidence, partially implantable HNS systems are underrepresented in this review, which limits the generalizability of the results. Significant heterogeneity was found for adverse event reporting. A framework for reporting HNS outcomes that includes AEs and side effects is proposed to facilitate comparability of the reported data.

## 1. Introduction

Obstructive sleep apnea (OSA) is a multifaceted disorder characterized by repeated upper-airway collapse during sleep, leading to intermittent hypoxia and frequent awakenings. It is associated with various symptoms and accompanying health conditions such as excessive daytime sleepiness, cognitive impairment, and an increased risk of traffic accidents [1]. Moreover, OSA is strongly linked to cardiovascular problems such as hypertension, coronary artery disease, and stroke, making it the most common modifiable cause of hypertension [2,3,4,5].

Over the decades, there have been significant advancements in understanding its prevalence, diagnostic methods, and treatment options. Initially defined in 1965, OSA treatment options were limited to surgical interventions like tracheotomy tube placement for bypassing airway obstruction or uvulopalatopharyngoplasty (UPPP), first proposed by Ikematsu, which became more popular in the same decade [6,7,8]. With the introduction of continuous positive airway pressure therapy (CPAP) in 1981, a crucial breakthrough in non-surgical management of OSA was marked [9]. Conservative medical measures, such as lifestyle changes, or physiological supports like CPAP or mandibular advancement devices (MADs) often have a slower onset of action, with potential side effects and complications becoming apparent as treatment progresses [10]. This is because they typically aim to treat the underlying cause of the problem and support or heal the body naturally, such as pneumatic splinting with CPAP therapy or mandibular advancement with an MAD. Because these approaches often take time to become fully effective, potential complications may develop gradually. This is especially true if the treatment does not have the desired effect or if there are unwanted side effects, such as pressure sores due to the need to fix the masks on the face, dry eyes due to air leakage, and so on. This requires longer and closer follow-up, not only to monitor that the desired positive effects of the therapy are achieved but also to avoid adverse events. On the other hand, complications or adverse events often occur earlier with interventional/surgical procedures because they involve direct intervention in the body and may be associated with direct risks during the procedure and in the immediate postoperative period [11]. These may include infection, bleeding, or other traumatic or implant-related events. Because these procedures have a faster and more direct effect on the body, complications may manifest more quickly, requiring careful monitoring and possibly intervention closer to the initial procedure. This makes the risks of interventional/surgical procedures immediately apparent and often makes them seem higher. Overall, however, it is important to carefully weigh the pros and cons of each type of therapy and decide which treatment option is most appropriate on an individual basis and considering recent evidence, the patient’s condition, the severity of the problem, and other relevant factors. Interventional/surgical therapies should not be considered inherently riskier.

This analysis aims to summarize and consolidate the best-available knowledge on the safety and adverse events of hypoglossal nerve stimulation for the treatment of OSA and provide a standardized format to display the evidence and allow for alternative interpretations.

## 2. Materials and Methods

### 2.1. Systematic Review

The research question was defined utilizing the PICO (Population, intervention, comparator, outcomes) format, centering on adult patients diagnosed with obstructive sleep apnea (OSA) who underwent hypoglossal nerve stimulation (HNS) therapy. The primary outcomes of interest encompassed the documented rates of adverse events and complications associated with HNS therapy, specifically focusing on the following clinical scenarios: all-cause mortality, HNS system explantation or replacement, re-operation or revision, bleeding or hematoma, postoperative pain, tongue abrasion, neuropraxia, and discomfort related to stimulation.

To identify pertinent publications, a comprehensive search strategy was developed, covering databases such as Medline, Embase, Cochrane, and Google Scholar (Table 1). The search was conducted up to 19 December 2023, and Rayyan software facilitated electronic data collection and screening [12].

Two researchers (MW and MB) independently conducted initial screening using a blinded approach to mitigate identification bias. Inclusion criteria encompassed studies with at least ten subjects published between January 2000 and December 2023, written in English, with a follow-up period of at least six months after implantation of the stimulation system, and reporting outcomes of interest as defined above. Exclusion criteria included review articles, case reports, animal or in vitro studies, editorials, abstracts, publications involving pediatric populations, and non-English language articles. Discrepancies in screening were resolved through discussion and documented following the Preferred Reporting Items for Systematic Review and Meta-Analysis (PRISMA) framework [13].

Full texts were reviewed to determine study eligibility guided by the PICO framework, which allowed the inclusion of both randomized controlled trials and observational cohort studies. Quality assessment employed the *Cochrane Handbook for Systematic Reviews of Interventions*, using the ROBINS-I tool for observational and case–control studies [14].

Upon identification of the final set of eligible articles, data on adverse events and complications were systematically extracted to an MS Excel database. Extracted information included details such as reported outcomes, number of subjects, reported follow-up period, study type, and device type used.

### 2.2. Statistical Analysis

Reported event rates, expressed as proportions of the study population affected by the respective events, were entered into RevMan 5.4 for meta-analysis. Pooled effect sizes were calculated from weighted average means with 95% confidence intervals. Heterogeneity among studies was assessed using the chi-squared test and Higgins and Thompson’s I2 statistics. Statistical significance was considered at an alpha level of 0.05 for two-tailed z-tests.

### 2.3. Standardized Definition of Clinical Endpoints

Based on the systematic review and identified clinical endpoints relevant to the safety profile of HNS therapy, a standardized set of therapy-related adverse events and side effects is proposed.

## 3. Results

### 3.1. Study Selection

**Figure 1:**
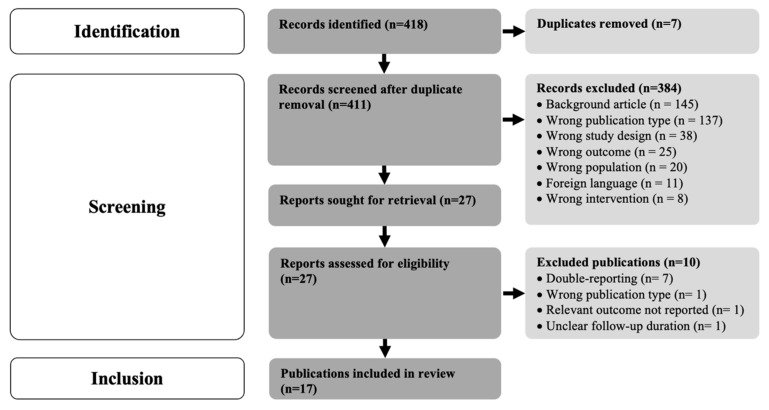
PRISMA flowchart of systematic literature search and review of identified studies.

Out of 418 articles initially screened, 27 underwent a thorough review for eligibility. A total of 384 studies were removed for various reasons, and 10 had to be excluded from further analysis after full-text review for eligibility, mainly due to reporting of study data already considered in other publications. Subsequently, 17 studies, collectively comprising data from 1962 patients and covering an average follow-up duration of 17.5 ± 16.9 months, met the inclusion criteria for further analysis (Figure 1). Most of these studies were found to have a low-to-moderate risk of bias (Table 2). Reporting of adverse events (AEs) across the included studies demonstrated notable heterogeneity, both in the utilized classifications or definitions, as well as the extent of reporting. Standardized reporting guidelines for AEs and complications with HNS therapy have not yet been established to the authors’ knowledge.

### 3.2. All-Cause Mortality

The all-cause mortality rate of patients receiving HNS therapy, calculated over a mean follow-up duration of 17.5 ± 16.9 months, was low, with a weighted average of 0.01% (95% CI = 0.00 to 0.2%). Further analysis revealed that reasons for death during therapy were cancer (n = 3), cardiac events (n = 3), and homicide (n = 1), and none was considered by the reporting researchers as related to HNS treatment.

### 3.3. HNS System Survival

**Figure 2:**
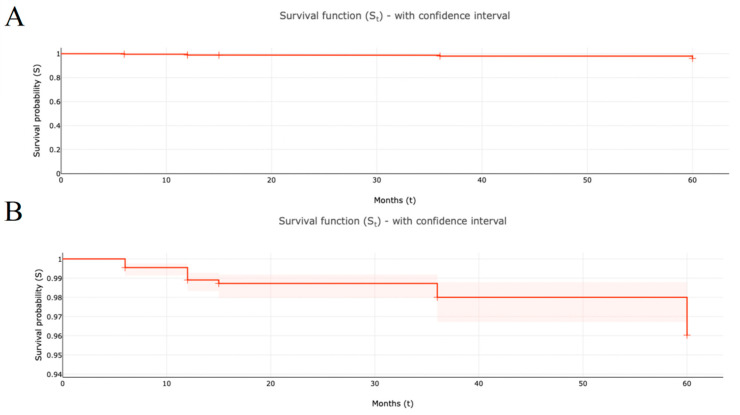
HNS system survival probability over follow-up time of included studies (S_t_ with 95% confidence interval): (**A**) = survival probability scaled from 0.0 to 1.0; (**B**) = survival probability scaled from 0.94 to 0.99.

A Kaplan–Meier analysis was conducted to evaluate device survival over the follow-up reported in included studies, which included all 1962 patients in the studies identified (Figure 2 and Table 3). At a median follow-up duration of 12.0 months, the system survival probability was 0.9890 (95% CI = 0.9833 to 0.9927) and 0.9834 at the maximum follow-up duration of 60 months (95% CI = 0.9768 to 0.9882.). The most common reasons for device explantation were infection in the implant area of the HNS system and patient request for various reasons.

### 3.4. Device- or Implantation-Related and Treatment-Related Adverse Events and Side Effects

**Figure 3:**
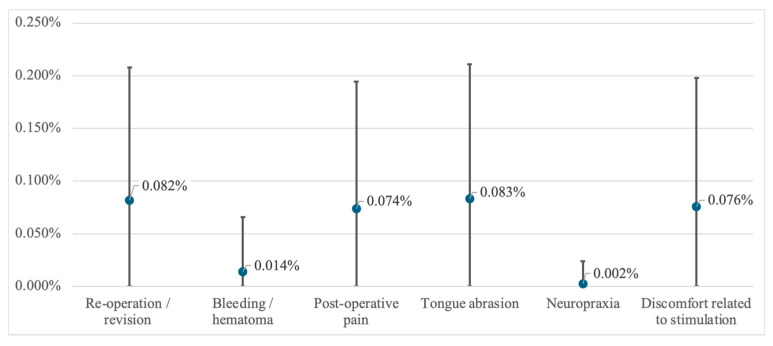
Weighted average rates and 95% confidence intervals of adverse events and side effects identified from included studies.

Rates of adverse events and side effects identified from the included studies and the respective 95% confidence intervals are presented in Figure 3. The pooled rate of surgical revision or re-operation was observed to be 0.08% (95% CI = 0.0 to 0.21%). Reasons identified encompassed revisions of hematoma or seroma, scar corrections, or repositioning of device components due to discomfort or technical dysfunction. Bleeding was defined as an event occurring either during surgery or leading to additional surgical measures, and occurred at a weighted average rate of 0.01% (95% CI = 0.00 to 0.07%). Neuropraxia was reported at a weighted average rate of 0.004% (95% CI = 0.00 to 0.02%), while postoperative pain was present at a rate of 0.07% (95% CI = 0.00 to 0.19%).

Most treatment-related side effects were reported as transient in the studies identified, and occurred primarily during the acclimatization period, while stimulation settings were being optimized over a period of three to six months after the implantation procedure. Discomfort related to stimulation was reported at a pooled rate of 0.08% (95% CI = 0.00 to 0.20%), while tongue abrasions were reported at a rate of 0.08% (95% CI = 0.00 to 0.21%).

### 3.5. Proposed Standardized Safety Endpoints for HNS Outcome Research

As a result of this systematic review, the absence of a standardized set of safety endpoints to be reported in clinical research on the outcomes of HNS therapy became apparent. This represents a significant gap, which complicates aggregation of results and makes comparability of outcomes difficult. To support future research and facilitate standardized data reporting, a set of relevant clinical endpoints are proposed and defined (Table 4). These endpoints shall be reported aside from the common objective and subjective efficacy and effectiveness endpoints relevant to the changes in OSA severity.

## 4. Discussion

To the best of the authors’ knowledge, this is the first study to report aggregated data on the safety profile of HNS therapy for the treatment of OSA in adults. Overall, adverse events and side effects with HNS treatment are occurring at relatively low frequencies and severe complications are rare. Given the invasive nature of HNS therapy, which requires surgical implantation of a neurostimulation system that can consist of different components that are placed on different parts of the body, the safety profile is positive.

Various studies have shown that HNS therapy is an effective treatment for moderate–severe OSA in selected adult patients who have difficulty accepting or adhering to CPAP treatment and leads to a high surgical success rate with reasonable long-term complication rate related to the implanted device [32]. Besides the effectiveness in reducing OSA disease severity, the treatment leads to substantial and sustained improvement in quality of life and is associated with a positive patient experience [33,34]. As with any other medical treatment, HNS is not free from adverse events or complications, as shown in this research. Earlier studies reported different causes of adverse events related to HNS treatment, which include sensor lead malfunction, with reports of pain and malfunction due to sensor tip separation and migration of implant components [35]. Additionally, adverse events such as pneumothorax and pleural effusion have been reported during HNS implantation, especially in patients with comorbidities [36]. Explantations of HNS systems have been rare, with reasons including necessity for an MRI at a time when the device was not compatible, cosmetic reasons, and device-related infections. These findings highlight the importance of considering potential complications and risks associated with HNS therapy when managing patients with OSA and underlines the importance of standardized reporting of surgical interventions for the treatment of OSA to allow informed decision-making.

Importantly, the overall mortality with HNS therapy is very low, and no deaths in the included studies were related to the treatment. Since OSA itself is associated with significant long-term mortality, the aggregated rate reported here is quite low. Since current practice often excludes patients with significant comorbidities from HNS therapy, this might have contributed. On the other hand, effective OSA treatment with HNS may a have a positive effect on overall mortality. With a follow-up time of 60 months, the included studies will likely be too short to document such an effect. In addition, none of the studies was powered to detect any effects on this endpoint.

A recent study by Moroco et al. evaluated device-related revision and explantation rates with HNS implants, using post-market surveillance data to calculate event rates and risks [37]. Overall, 20,881 HNS treatments included, explantation and revision rates within the first year were 0.7% and 1.5%, respectively. In this real-world data set, the most common indication for explantation procedures was infection (0.4%) and surgical correction for revision procedures (0.7%). For HNS system with three-year follow-up data post-implantation (n = 5820), the explantation rate was 2.7% and the revision rate was 3.5%. Elective removal was the most common indication for explantation during this period (1.5%), while surgical correction was the most common reason for revision (1.1%). This study concluded that HNS demonstrates efficacy comparable to clinical trial data, with low rates of explantation and revision, indicating a satisfactory safety profile for this technology in real-world settings.

Thaler and Schwab reported on their experience implementing an HNS program at a tertiary care center [38]. They emphasized the importance of a team-based approach to patient flow, which requires close collaboration between sleep physicians and surgeons. The process included steps such as patient selection, preoperative evaluation, device implantation and activation, and long-term follow-up. The authors also discussed the challenges of patient selection for HNS treatment and emphasized the importance of thorough training and surgical planning. Another study from Murphy et al. found that surgical time for HNS implantation decreased significantly after the first five implants and then stabilized [39]. The average surgical time reduced by 27% from the first to the fifth implant, but the study did not determine when the learning curve reached a plateau. Adverse events were mostly resolved without intervention, and there were no serious complications related to the procedure. Interestingly, in this evaluation, surgeon experience did not significantly decrease the rate of surgical complications, possibly due to the low overall rate of serious complications and the limited number of implants at some centers.

Researchers at Virginia Commonwealth University conducted a study in 2022 to compare the safety of different surgical procedures for OSA based on real-world data from a federal health record database of sleep surgery outcomes [40]. One of the main findings from that study was that HNS statistically had a much lower complication rate (2–3%) compared to palatal surgery (20%) and multilevel surgery (21%) and lower readmission rates in the immediate postoperative period. The 90-day readmission rate was lowest in the HNS cohort, with 4% compared to palatal surgery alone (11%) and multilevel surgery (12%). The most common reasons for complications after traditional sleep surgery were bleeding, infection, wound dehiscence, respiratory compromise, myocardial infarction, stroke, deep vein thrombosis, renal failure, and pneumonia for palatal surgery. Specific complications reported for HNS procedures included the need for device revision, tongue weakness, difficulty speaking or swallowing, and device-related pain.

A potential explanation for the low rate of adverse events and complications from HNS therapy is the extent of standardization of patient pathways and surgical technique, which were optimized during clinical validation. Those were implemented in a relatively consistent approach, for example, via educational measures. In Germany, for example, a position paper from the German Society of Oto-Rhino-Laryngology, Head and Neck Surgery defines requirements for provider infrastructure and processes to ensure outcome quality and minimize complications, as well as minimal reporting standards to document treatment outcomes [41].

HNS therapy has been established as a viable alternative for the treatment of OSA over the past decade, offering significant and sustained improvements in disease control and quality of life. This systematic review underscores the favorable safety profile of HNS, highlighting its potential as a reliable treatment option. The findings have several important implications for clinical practice. Though the overall rate of complications and adverse events is low, patients should be thoroughly informed about the nature of HNS therapy, including the surgical procedure, potential risks, and benefits. Emphasis should be placed on discussing the possibility of temporary stimulation-related discomfort during acclimatization and other minor side effects during treatment initiation. While the surgical revision rate is low, clinicians should be prepared and trained to address any complications that may arise, such as device-related infections or explantation. For patient care after implantation, strategies should be developed to manage common side effects like tongue abrasions and stimulation-related discomfort, ensuring long-term patient comfort and sustainable therapy adherence.

### Limitations

This study, though performed to high methodological standards and conducted with scientific rigor, is not free from limitations. It is important to underline that the extent of reporting adverse events among the identified research is low. After completion of the literature search process, a few relevant studies were published, which shed more light on this topic and confirm the findings from this analysis. Additionally, a high heterogeneity in reporting was found in published research on HNS outcomes. As such, among the 17 studies included, each varied with regard to the clinical endpoints reported. A set of standardized clinical safety endpoints as proposed in this article may help to increase the extent and consistency of reporting and improve comparability of outcomes. It is further important to note that most included studies reported data on a unilateral, breathing-synchronized HNS device, which may limit the generalizability of the results to other stimulation methods.

Furthermore, it is important to acknowledge that only two studies included were found to have low overall bias. As such, multiple confounders and biases could be present in the underlying data, which may have an impact of the findings presented in this study. Given the heterogeneity in reporting mentioned before, it was not possible to correct aggregated data, e.g., for device settings, medical service-procedures, and other parameters, related to the care context. For the endpoints we report here, such as explantation or re-operation, we believe that the data included are strong enough to conclude that HNS is overall a relatively safe surgical treatment option.

The findings of this study highlight the different types of adverse events and complications and the rate at which they can occur. The study also sheds light on the relatively high degree of heterogeneity in reporting these events in clinical studies and the need for standardization of endpoints. Given the high rates of complications and the irreversibility of traditional OSA surgery, this may also be relevant to this segment of OSA treatments. Improving reporting of adverse events will ultimately lead to a better understanding of potential risks related to surgical treatment for OSA, which will enable providers and patients to make informed decisions that consider individual characteristics and preferences of the patient. The set of endpoints related to adverse events and complications with HNS therapy proposed in this article may help to improve the database for this treatment and allow comparability of results from different care settings and across the spectrum of surgical interventions for OSA.

## 5. Conclusions

This review summarizes patient safety in the surgical intervention of HNS treatment that involves using an implantable neurostimulation system. The pooled mortality rate associated with HNS therapy was 0.01%, with reported deaths unrelated to the treatment. The HNS system safety profile was positive, with a device survival probability of 0.9834 (95% CI = 0.9768 to 0.9882) over a follow-up time of 60 months and a pooled surgical revision rate of only 0.08%. Common treatment-related side effects included transient stimulation-related discomfort and tongue abrasions, which mainly occurred during the acclimatization phase. Heterogeneity in reporting safety-related events was high across the included studies, which led to the development of a standardized set of endpoints, which is proposed in this article to harmonize safety data related to HNS therapy.

## Figures and Tables

**Table 1:** Search strategy for identification of relevant literature.

Keywords	Publication Date	Inclusion Criteria
Obstructive sleep apnea [Title/Abstract] AND (Implantable nerve stimulator [Title/Abstract] OR hypoglossal [Title/Abstract] OR Inspire implant [Title/Abstract])	January 2000–December 2023	Publication in English language Reporting a follow-up period of at least six months after implantation Reporting follow-up data of at least ten subjects All-cause mortality, HNS system explantation or replacement, re-operation or revision, bleeding or hematoma, postoperative pain, tongue abrasion, neuropraxia, and discomfort related to stimulation.

**Table 2:** Included studies, study characteristics, and risk-of-bias assessment using the ROBINS-I tool [14].

Study	Year	Sample Size	Follow-Up (Months)	Death	Explant/ Replacement	Re-Operation/ Revision	Bleeding	Post-Op Pain	Tongue Abrasion	Neuropraxia	Discomfort	Study Design	Device Type	Overall Bias	Reference
Ali NS et al.	2023	215	60	X	X	X	X	X	X	X	X	Observational cohort study	Fully implantable	● critical	[15]
Eastwood PR et al.	2011	21	6	X	X	X	X	X	X	X	X	Observational cohort study	Fully implantable	● serious	[16]
Eastwood PR et al.	2019	27	6	X	X	X	X	-	X	-	X	Observational cohort study	Partially implantable	● moderate	[17]
Friedman M et al.	2016	43	6	X	X	X	X	X	X	X	X	Observational cohort study	Fully implantable	● moderate	[18]
Heiser C et al.	2017	31	12	X	X	X	X	X	X	X	X	Observational cohort study	Fully implantable	● moderate	[19]
Hinder D et al.	2022	50	12	X	X	X	-	-	-	-	-	Observational cohort study	Fully implantable	● moderate	[20]
Kezirian EJ et al.	2014	32	12	X	X	X	X	X	X	X	X	Observational cohort study	Fully implantable	● moderate	[21]
Mwenge GB et al.	2013	14	12	X	X	X	X	-	-	-	-	Observational cohort study	Fully implantable	● moderate	[22]
Patil R et al.	2021	53	12	X	X	X	X	X	X	X	X	Observational cohort study	Fully implantable	● moderate	[23]
Sarber KM et al.	2020	18	6	X	X	X	X	X	X	X	X	Observational cohort study	Fully implantable	● serious	[24]
Schwartz AR et al.	2023	138	15	X	X	X	X	X	X	X	X	Randomized-controlled study	Fully implantable	● moderate	[25]
Steffen A et al.	2019	60	36	X	X	X	X	X	X	X	X	Observational cohort study	Fully implantable	● moderate	[26]
Suurna M et al.	2021	1019	12	X	X	X	X	-	X	-	X	Observational cohort study	Fully implantable	● moderate	[27]
Van de Heyning PH et al.	2012	28	6	X	X	X	X	X	X	X	X	Observational cohort study	Fully implantable	● moderate	[28]
Veugen CCAM et al.	2023	25	12	X	X	X	X	X	X	X	X	Observational cohort study	Fully implantable	● low	[29]
Woodson BT et al.	2018	126	36	X	X	X	X	X	X	X	X	Observational cohort study	Fully implantable	● low	[30]
Zhu Z et al.	2018	62	12	X	X	X	X	X	X	X	X	Observational cohort study	Fully implantable	● moderate	[31]

**Table 3:** HNS system survival probability over follow-up time of included studies (SE = standard error of survival probability, CI = confidence interval of survival probability).

Month	Explantation Events	Subjects at Risk (n)	Censored Subjects at End of Period	Survival Probability (S_t_)	SE	Lower 95% CI	Upper 95% CI
0	0	1962	0	1.0000	0.0000	1.0000	1.0000
6	9	1962	137	0.9950	0.0016	0.9907	0.9973
12	12	1825	1286	0.9890	0.0023	0.9833	0.9927
15	1	539	138	0.9885	0.0024	0.9827	0.9923
36	3	401	60	0.9870	0.0025	0.9809	0.9911
60	7	342	341	0.9834	0.0029	0.9768	0.9882

**Table 4:** Proposed standardized clinical endpoints for safety data in HNS therapy outcome research.

Proposed Clinical Endpoint	Timing/Definition
1. HNS system survival	End of study follow-up period A: Initially implanted HNS system in situ B: HNS system explanted upon patient request
2. Re-operation/revision	1. Re-operation or surgical revision in the index admission or ≤30 days of the index procedure A: Re-operation or surgical revision due to bleeding, hematoma, or seroma B: Re-operation or surgical revision related to any component of HNS system C: Re-operation or surgical revision not related to any component of HNS system (hard- or software)
	2. Re-operation or surgical revision > 30 days after the index procedure A: Re-operation or surgical revision due to bleeding, hematoma, or seroma B: Re-operation or surgical revision related to any component of HNS system C: Re-operation or surgical revision not related to any component of HNS system (hard- or software)
3. Postoperative infections	Intervention due to infections ≤ 60 days of the index procedure A: Re-operation or surgical revision due to infection in the implant area B: Explant of any component of HNS system due to infection in the implant area
4. Neuropraxia or swallowing difficulties	Neuropraxia or swallowing difficulties presenting after the implantation procedure A: Resolving without sequelae within 3 days after implantation procedure B: Resolving without sequelae within 7 days after implantation procedure C: Resolving without sequelae within 14 days after implantation procedure D: Adverse event with sequelae or presenting > day 14
5. Post-operative pain	Post-operative pain related to any medical measure related to the implantation procedure A: Pain requiring NSAR or other non-opioid pain-medication B: Pain requiring oral opioids C: Pain requiring intravenous pain medications D: Pain requiring re-operation or surgical revision
6. Discomfort related to stimulation	1. Discomfort related to HNS stimulation occurring ≤ 30 days after system activation A: Discomfort not impacting HNS adherence B: Discomfort impacting HNS adherence
	2. Discomfort related to HNS stimulation occurring > 30 days after system activation A: Discomfort not impacting HNS adherence B: Discomfort impacting HNS adherence

## Data Availability

The data that support the findings of this study are not openly available due to reasons of sensitivity and are available from the corresponding author upon reasonable request.
